# Helminth Immune Modulation and Invasive Fungal Infections in Sub-Saharan Africa

**DOI:** 10.3390/jof12020160

**Published:** 2026-02-23

**Authors:** Luis Fonte, Yaxsier de Armas, Héctor R. Pérez-Gómez, Enrique J. Calderón

**Affiliations:** 1 Centro de Investigación, Diagnóstico y Referencia, Instituto de Medicina Tropical “Pedro Kourí”, La Habana 11400, Cuba; 2 Departamento de Microbiología y Patología, Instituto de Patología Infecciosa y Experimental “Francisco Ruiz Sánchez”, Centro Universitario de Ciencias de la Salud, Universidad de Guadalajara, Guadalajara 44100, Mexico; yaxsier.dearmas@academicos.udg.mx; 3 Departamento de Anatomía Patológica, Instituto de Medicina Tropical “Pedro Kourí”, La Habana 11400, Cuba; 4 Instituto de Patología Infecciosa y Experimental “Francisco Ruiz Sánchez”, Centro Universitario de Ciencias de la Salud, Universidad de Guadalajara, Guadalajara 44100, Mexico; hrulito@hotmail.com; 5 Instituto de Biomedicina de Sevilla, Hospital Universitario Virgen del Rocío/Consejo Superior de Investigaciones Científicas/Universidad de Sevilla, 41013 Sevilla, Spain; 6 Centro de Investigación Biomédica en Red de Epidemiología y Salud Pública (CIBERESP), 28029 Madrid, Spain

**Keywords:** sub-Saharan Africa, helminth immune modulation, HIV infection, tuberculosis, Invasive Fungal Infections

## Abstract

Sub-Saharan Africa, a region marked by enormous social and health inequalities, has the largest population infected with HIV and *Mycobacterium tuberculosis*, which are considered the main risk factors for fungal infections. At the same time, sub-Saharan Africa is the region of the world with the highest rates of helminth infections, whose immunomodulatory effects impair the host’s immune responses to other microorganisms, including HIV and *M. tuberculosis*. Through this indirect way, helminth immune modulation could be another syndemic factor influencing the development of fungal infections. However, some epidemiological peculiarities of five fungal diseases in sub-Saharan Africa, which we analyze in this paper, suggest that the influence of helminth immune modulation on the development of fungal infections there could also be direct. In light of the knowledge of all those interactions, any healthcare and epidemiological approach to Invasive Fungal Infections in sub-Saharan Africa should be carried out from a syndemic perspective that takes into account the ways in which social environments contribute to the clustering of infections, the pathways through which infecting microorganisms could interact biologically in each individual, influencing the development and evolution of the disease in course, and the ways in which those interactions complicate diagnosis, treatment, and control.

## 1. Introduction

The clinical spectrum of human fungal infections is broad, ranging from asymptomatic superficial forms to systemic diseases due to deep tissue invasion [1]. The term Invasive Fungal Infections (IFIs) encompasses the systemic infections resulting from the establishment of fungi in deep tissues [2]. The occurrence of IFIs depends on the species involved and the host immune competence. In fact, most IFIs are produced by fungal species which only cause diseases in persons who suffer from some types of immunodeficiency, such as those related to other infections (human immunodeficiency virus—HIV- infection, tuberculosis), neutropenia, cancer, and immunosuppressive chemotherapy [2].

Since the 1950s, a decade marked by the beginning of the use of immunosuppressive and antineoplastic therapies, there has been a continuous increase in the global burden of human fungal diseases [3]. Current worldwide statistics show that around 6.5 million people develop an IFI each year [4]. These fungal infections are directly responsible for about 2.55 million deaths annually, surpassing those caused by malaria and tuberculosis [4,5].

Despite the global advancements in the use of traditional procedures and in the development of new methodologies for the detection of fungal infections, the available information regarding mycosis in Africa, particularly in the sub-Saharan region, is insufficient. The primary obstacle to acquiring that data, and therefore reaching a clearer comprehension of the epidemiological peculiarities of fungal diseases in Africa, has been the limited accessibility of diagnostic resources there [6]. It is estimated that 47 million people experience a fungal disease there each year, of which around 1.7 million suffer from an IFI [7].

In 1999, Casadevall and Pirofski proposed the Damage Response Framework for Microbial Pathogenesis (DRF for Microbial Pathogenesis), which defines microbial pathogenesis as an outcome resulting from the interplay between a host and a pathogen [8]. This model, which has been used to achieve a better understanding of complex pathogenic processes, such as cryptococcosis, COVID-19 and talaromycosis [9,10,11], suggests that the clinical disease can arise from either uncontrolled spread of the pathogen or an adverse (too weak or too strong) immune response by the host, both resulting in host damage [8]. More recently, the development of the syndemic theory has provided complementary tools for a better understanding of the effects of ecological, social and biomedical factors on the co-occurrence and clustering of infectious diseases [12]. The syndemics approach examines why certain diseases cluster, the pathways through which they interact biologically in individuals and within populations, and the ways in which social and environmental conditions contribute to disease clustering and interaction [12,13].

Geographical distribution studies on helminth infections and other pathogens, particularly those of greater clinical and epidemiological significance, such as HIV and *M. tuberculosis*, have shown that these coexist in regions where socio-economic and environmental conditions favor their transmission [14,15,16]. However, another syndemic factor fosters this coexistence at biological level: infections by helminths stimulate the production of type 2 cytokines (IL-4, IL-5, and IL-13) and activate regulatory T mechanisms (Treg cells, IL-10, and TGF-β, among others) that, together, inhibit the development of Th1 and Th17 effector responses, necessary for the control of HIV and *M. tuberculosis* infections [14,16,17,18,19]. In addition, and in harmony with that observation, Th1 and Th17 CD4+ T cells are particularly important for the control of fungal infection [20]. Here, taking into account the theoretical frameworks mentioned above, we analyze a possible influence of helminth immune modulation on some epidemiological peculiarities of IFIs in Africa, particularly in the sub-Saharan region.

## 2. CD4+ Th1 and Th17 Cell Responses Are Critical for Resistance to Fungi

Thousands of species of fungi are present in nature, but only a relatively small number (around 500 species) cause infections in humans [20]. As Arturo Casadevall, in an enlightening analysis of the extraordinary resistance of human to fungal infections, summarized, “The remarkable resistance of immunologically intact hosts is believed to result from the combination of endothermy, which creates a thermal exclusion zone for many species of potentially pathogenic fungi (their viability decline above 30 °C), and adaptive immunity, which enhances the protection provided by innate immunity” [21].

The sensing of pathogen-associated molecular patterns (PAMPs) of the fungal cell wall by pattern recognition receptors (PRRs) in the membrane of innate immune cells is the first step in mounting antifungal immune responses [22,23]. After the detection of fungi, two series of innate immune mechanisms for microorganism clearance are triggered: on one hand, and ending in the intracellular compartment, polymorphonuclear neutrophils (PMNs), macrophages, and dendritic cells (DCs) undergo phagocytosis and killing of the invading fungus; on the other hand, and happening in the extracellular space, PMNs can release extracellular traps that capture hyphal forms of fungi, natural killer cells can directly kill fungi by secreting cytotoxic molecules, and proteins of the complement system can deposit on the fungal cell surface, mediate their killing directly and, at the same time, activate other immune cells [20,22,23].

DCs, which connect natural and adaptive immunity, process and present fungal antigens to CD4+ and CD8+ T cells, as well as secrete cytokines to shape naïve T cell differentiation [20,22,23]. CD4+ Th1 and Th17 T cells are critical for immunity against fungi, particularly against intracellular ones (*Cryptococcus* spp., *Histoplasma* spp.), as these lymphocytes secrete pro-inflammatory cytokines such as IFN-γ and IL-17 that recruit and activate phagocytes, including classically activated macrophages (M1), to eliminate the fungi [20,22,23,24]. CD8 T cells, which synthesizes IFN-γ too, control fungal infection by mediating specific effector functions like activating innate immune cells, lysing of infected host cells, and direct killing of pathogenic fungus [23,25].

The role of CD4+ Th2 T cells in immunity to fungi, with the exception of the limitation of inflammatory response to *Pneumocystis jirovecii* that we comment on below, is more associated with disease exacerbation [20,26]. IL-4 and IL-13 cytokines, released by Th2 CD4+ T cells, inhibit Th1 type differentiation, which results in the limitation of pro-inflammatory cytokine production, and promote the secretion of the anti-inflammatory and immunomodulatory cytokines IL-10 and TGF-β by surrounding alternatively activated macrophages [20,27]. Regulatory T cells (Tregs), also producing IL-10 and TGF-β, can suppress the inflammatory immune response that results in higher susceptibility to fungal infections [20,28].

Compared to cellular immune responses, the contribution of humoral immunity to the defense against fungal infections is less well known [20]. The presence of specific antibodies in the IFIs suggests that these are insufficient to prevent fungal infections [29]. Nevertheless, depletion of B cells in peripheral blood mononuclear cells with rituximab results in reduced Th17-mediated antifungal responses [30].

## 3. Helminths Down Regulate CD4+ Th1 and Th17 Cell Responses

Even though some types of sanitary interventions, such as educational and mass deworming programs, have had some success, helminth infections remain an important health problem in several areas of the planet [31,32]. Sub-Saharan Africa (SSA) remains the region of the world with the highest rates of helminth infections, particularly soil-transmitted helminths infections and schistosomiasis [31,32,33]. For example, of the world’s 207 million estimated cases of schistosomiasis, 93% occur in SSA (192 million) [31]. In endemic areas, the prolonged coevolution of humans and helminths has led to the development of defensive responses by the former and to the achievement of complex immune modulatory means by the latter. To control helminth infections, adaptive immunity of the host usually develops type 2 immune responses, including Th2 cell and cytokines release such as IL-4, IL-5 and IL-13 [17]. This host–helminth interaction has, at least, two additional outcomes: (i) the classical and best-known down-regulation of type Th1 [17,18] and type Th17 responses (and its related cytokines IL-12, IFN-γ, IL17, IL-23, and TNF-α) by the Th2 cytokines, and (ii) the relatively less-known down-regulation of immune responses by enhancing FOXP3+ T regulatory cells, B regulatory cells, and M2 macrophages activities, which together cause the release of regulatory cytokines such as IL-10 and transforming growth factor [19].

Because of their immunomodulatory effects, helminths can limit the development of defensive inflammatory responses during infection by viruses and bacteria. Some examples include the following: (i) In HIV-infected individuals, deworming improved the CD4+ counts and decreased the viral load [15]. (ii) Mice infected by *Nippostrongylus brasiliensis* showed increased susceptibility to *M. tuberculosis*. Apparently, M2 macrophages with impaired killing capacity in a less inflammatory Type 2 pulmonary milieu function as a mycobacteria reservoir [34]. (iii) A lower production of IFN-γ and higher production of IL-10 has been observed in peripheral blood cultures of patients co-infected with helminths and *M. tuberculosis*, compared to patients infected only with the mycobacteria [35].

## 4. Helminth Immune Modulation May Influence Immune Response to Fungus

Africa, particularly the sub-Saharan region, continues to have the highest population infected with HIV and *M. tuberculosis*, which are considered the major risk factors for the development of fungal infections [36,37,38]. Coincidentally, SSA is the region of the world with the highest rates of helminth infections [31,32]. In addition to the common socioeconomic predisposing factors present there, it has been demonstrated that helminth immunomodulation impairs the host immune response to HIV and *M. tuberculosis*, increasing the severity of the infections caused by those microorganisms [15,34,35]. Through this indirect way, helminth infections could be another syndemic risk factor influencing the development of fungal infections. However, some epidemiological peculiarities of, at least, five fungal diseases in SSA suggest that the influence of helminth infections on the development of fungal infections there could also be direct:

I. Cryptococcosis, caused by the intracellular fungus *Cryptococcus* spp., is one of the most prevalent and dangerous mycoses in SSA [38]. In 2014, it was calculated that, out of 223,100 worldwide cases of cryptococcal meningitis, 162,500 (73%) occurred in the sub-Saharan area; and, out of 181,100 global deaths due to this fungal disease, 135,900 (75%) happened in that region [39]. After the sensing of PAMPs of *Cryptococcus* spp. cell wall by PRRs on the membrane of innate immune cells, signal transduction activates the effector functions of those cells, mainly phagocytosis and the generation of inflammatory response mediators such as cytokines, fungicidal compounds, and acute phase reactants [40]. These activities can result in the clearance of the fungus or in the development of adaptive immune responses, in which CD4+ Th1 and Th17 T cells, CD8+ T cells and B cells are the major immune cell types involved [41]. Acting synergistically, these cells trigger cytokine-mediated activation of effector cells, including classical macrophages, direct cellular cytotoxicity, and antibody-dependent defensive mechanisms [41]. Nevertheless, resistance to *Cryptococcus* spp. infection is primarily dependent upon CD4+ Th1 and Th17 T cells, which secrete IFNγ and other inflammatory mediators [38]. This particularity suggests that helminth infections, which are also more prevalent in SSA and down-regulate Th1 and Th17 type responses, could be an additional reason for the higher prevalence and severity of cryptococcal infection in that African region.

II. Histoplasmosis, which can develop after inhaling spores of the dimorphic fungus *Histoplasma capsulatum* (*var. capsulatum* and *var. duboisii*), is a serious fungal disease endemic in several parts of the world, including SSA [42]. Upon inhalation, fungal spores settle into bronchioles and alveoli and convert into pathogenic yeasts within resident phagocytes and DCs [43]. These cells transport *Histoplasma* to visceral and lymphoid organs where the infection expands. Within few weeks, Th1 immunity is activated, arresting *Histoplasma* replication in monocytes and macrophages [43]. On the contrary, increased Th2 responses exacerbate disease by producing type 2 cytokines that reduce the microbicidal activities of macrophages [43]. In SSA, HIV infection is considered the greatest risk factor for histoplasmosis in the adult population, mainly for its disseminated form [44]. However, HIV infection does not seem to be a predominant risk factor in the pediatric population. Ekeng et al., in a comprehensive review of the literature on histoplasmosis in pediatric patients, highlighted 44 cases of histoplasmosis among children in SSA. Despite 56.8% of the cases corresponding to disseminated histoplasmosis, HIV infection was only reported in 6.8% of them [45]. Alternatively, histoplasmosis in the SSA pediatric population has been associated with other risk factors, including environmental exposures, malnutrition, malignant disorders, immunosuppressive therapies, and immunodeficiencies [46,47]. We opine that helminth infections, which are highly prevalent in sub-Saharan children and down-regulate Th1 and Th17 immunity while enhancing Th2 responses, may be an additional risk factor for *Histoplasma* infection in pediatric populations there.

III. *Pneumocystis* pneumonia (PcP), also known as pneumocystosis, is caused by *Pneumocystis jirovecii* [48]. *Pneumocystosis*, characterized by an intense pulmonary inflammatory reaction that can lead to death, is one of the most common fungal diseases in immunosuppressed individuals [48,49]. During the 1980s, it was estimated that two-thirds of the HIV-infected people worldwide developed PcP [50]. Paradoxically, studies conducted in those years in some SSA countries with a high prevalence of HIV infection reported that PcP was less common [51,52,53]. Although the worldwide prevalence of pneumocystosis has declined since the introduction of cotrimoxazole prophylaxis and active antiretroviral therapy during the 1990s, PcP remains the most important opportunistic infection defining AIDS in the United States and Europe [48,54]. Paradoxically again, in SSA after the administration of the aforementioned drugs, mortality in people infected with HIV is dominated by other infectious diseases, mainly tuberculosis [54]. Some factors, or a combination of them, have been alluded to explain the lesser severity of PcP in the sub-Saharan region: differences in PcP diagnosis methodologies [50,54,55], lesser virulence of circulating strains of *P. jirovecii* [56], higher host resistance to *P. jirovecii* infection [57], elevated ambient temperature [58], and higher rates of bacterial pneumonia and tuberculosis in HIV-infected African adults [50,54].

Considering the immunomodulatory effects of helminth infections described before in this document, a complementary explanation for the apparently lower severity of PcP in SSA is possible: the inhibition of Th1 and Th17 type responses as a consequence of regulatory mechanisms induced by helminth infections could be limiting the intense pulmonary inflammatory reaction to *P. jirovecii* infection [59]. Observations in rodents, the animal model of PcP, provide an additional support to this hypothesis: although macrophages of M1 and M2 profiles can phagocyte *Pneumocystis*, those that harbor an alternative activation M2 profile (that is, activation through cytokine of the Th2 response) are preferentially involved in immunocompetent mice. This M2 profile, which could be related to the mechanisms of helminth immune modulation, is consistent with the predominant Th2 profile reported in immunocompetent mice infected with Pneumocystis [60,61]. In line with this hypothesis, it is interesting to mention that the severity of COVID-19 (Coronavirus Disease 2019), another respiratory disease related to the development of an intense pulmonary inflammatory reaction, is lower in SSA compared to Europe and the United States [62].

IV. The inhalation of conidia of several species of *Aspergillus* can result in two well-defined clinical forms of aspergillosis: invasive aspergillosis (IA) and allergic bronchopulmonary aspergillosis (ABPA) [38]. IA, which affects more than 300,000 people worldwide each year, is a progressive and debilitating parenchymal lung disease occurring in patients with underlying lung conditions and deficiencies in the innate immune system, particularly in neutrophil phagocytic activity that prevents conidia germination [38,63,64,65]. On the other hand, ABPA is a severe inflammatory reaction caused by a Th2 hyperresponse to *Aspergillus* antigens [66]. That is, although myeloid cells are essential for protection against *Aspergillus*, and their deficient functioning can lead to IA, fungus-specific CD4 Th2 cells contribute to *Aspergillus*-associated allergic disease [65,66]. The incidence of patients with asthma is growing in Africa and, based on the findings of some recent studies, fungal sensitization has been observed among asthmatic populations. In a systematic review of studies conducted in this continent, the prevalence of fungal sensitization (positive type-1 skin prick test to *Aspergillus* allergens) was high in the asthmatic groups, with an average of 28% [67]. In Uganda, a country with a high prevalence of Th2-polarizing helminth infections, a very high positivity (60%) of *A. fumigatus* skin test was found in apparently healthy non-atopic individuals [68]. Interestingly, that positivity was higher in younger population groups, which are typically the individuals most infected by helminths [68].

V. Emergomycosis, a severe multi-organ disease more commonly observed in Africa (74% of global cases), can develop after inhaling conidia of the dimorphic fungus *Emergomyces* spp. present in soil [69,70]. Once inhaled, *Emergomyces* conidia settle in the terminal regions of the bronchiolar tree, where they presumably undergo a morphological transition to budding yeasts capable of extrapulmonary dissemination in susceptible hosts [71]. Based on molecular phylogenetic analyses, seven species of the genus *Emergomyces* have been identified [69]. Among them, *E. pasteurianus* is the most widespread species, while *E. africanus* is endemic to Southern Africa, where the fungus has been primarily detected in patients with advanced HIV disease (median CD4+ T cell count 16 cells/μL) and extensive cutaneous lesions [70,71]. Little is known about the pathogenesis and the host’s immune response to *Emergomyces* spp. An increase in pro-inflammatory cytokine production was detected in homogenized lung supernatants of mice infected with *E. africanus* [72]. In addition, infected Rag-1-deficient mice, lacking mature T-and B-cells, shown an increased fungal burden associated with reduced IFN-γ production [72]. Taken together, these data suggest that hosts develop a protective type-1 immune response to control *Emergomyces* infection.

Some factors, or combinations of them, have been mentioned to explain the increase in reports of emergomycosis in South Africa over the past two decades: the high prevalence of HIV-infected individuals and non-adherence to antiretroviral therapy, the high spore load of *Emergomyces* species in the region, and, most consistently, the improvement in diagnostics and the identification of fungi within the national health system, among others [69,73]. The long-distance dispersal of airborne *E. africanus* vegetative structures [74], the frequent observation of skin lesions in HIV-positive individuals in care centers of sub-Saharan countries [73], the limited infrastructure and clinical expertise for the diagnosis of emergomycosis there [70], and the potential syndemic influence of other infectious diseases, such as helminth infections, suggest that this fungal disease may be a health problem in SSA.

## 5. Conclusions

SSA, a region marked by enormous social and health inequalities, has the largest population infected with HIV and *M. tuberculosis*, which are considered the main risk factors for fungal infections [7,38,75]. Coincidentally, SSA is the region of the world with the highest rates of helminth infections, whose immunomodulatory effects impair the host’s immune responses to other microorganisms [31,32,34,35]. Recent advances in the knowledge of immunity to HIV, *M. tuberculosis*, fungi, and parasites, including a better understanding of the mechanisms by which helminths modulate immune responses, are allowing a better comprehension of some peculiarities of IFIs in SSA. In line with this, and as has already been suggested for some coendemic infectious diseases in other settings [63], all healthcare and epidemiological approaches to IFIs in SSA should be done from a syndemic perspective that takes into consideration the ways in which social environments contribute to infections clustering, the pathways through which infecting microorganisms could interact biologically in each individual, influencing the development and evolution of the disease in course, and the ways in which those interactions complicate diagnosis, treatment and control.

## Data Availability

Not applicable.

## Abbreviations

The following abbreviations are used in this manuscript:

IFIs	Invasive Fungal Infections
HIV	Human Immunodeficiency Virus
PAMPs	Pathogen-Associated Molecular Patterns
PRRs	Pattern Recognition Receptors
PMN	Polymorphonuclear Neutrophils
DCs	Dendritic Cells
SSA	Sub-Saharan Africa
COVID-19	Coronavirus Disease 2019
IA	Invasive Aspergillosis
ABPA	Allergic Bronchopulmonary Aspergillosis
PcP	Pneumocystis pneumonia
